# Prediabetes in rural adolescent girls from DERVAN cohort: data from the KONKAN region of the state of Maharashtra, India (DERVAN-4)

**DOI:** 10.3389/fpubh.2023.1181401

**Published:** 2023-08-03

**Authors:** Suvarna Patil, Netaji Patil, Pallavi Hardikar-Bhat, Omkar Dervankar, Charudatta Joglekar, Rohit Bhat, Ajit Nandoskar, Arvind Yadav, Anup Nilawar

**Affiliations:** ^1^Department of Medicine, BKL Walawalkar Rural Medical College, Sawarde, Maharashtra, India; ^2^Department of Radiology, BKL Walawalkar Hospital and Rural Medical College, Sawarde, Maharashtra, India; ^3^Regional Centre for Adolescent Health and Nutrition, BKL Walawalkar Rural Medical College, Sawarde, Maharashtra, India; ^4^Department of Biochemistry, BKL Walawalkar Rural Medical College, Sawarde, Maharashtra, India

**Keywords:** prediabetes, adolescents, rural, undernutrition, India

## Abstract

**Background:**

India is witnessing an epidemic of type 2 diabetes. Overweight/obesity, overnutrition, physical inactivity, and family history are well-known risk factors for diabetes. We investigated the role of undernutrition in the development of diabetes among rural adolescent girls.

**Methods:**

DERVAN cohort study was set up in the KONKAN region of the western Indian state of Maharashtra. It enrolled 1,520 adolescent girls (16–18 years old at the time of enrollment). We measured glycemic parameters (glucose, insulin, and HbA_1_C) and body size using anthropometry and body composition using bioimpedance. Prediabetes was diagnosed using the American Diabetic Association (ADA) criteria. We also calculated various HOMA indices for insulin resistance (HOMA-IR), β-cell function (HOMA-β), insulin sensitivity (HOMA-S), and compensatory β-cell response using a homeostasis model. BMI, body fat%, and waist circumferences were treated as exposures and all the glycemic parameters and indices as outcomes.

**Results:**

The median age of the subjects was 16.6 years. The median weight, height, and BMI were 40.7 kg, 151.7 cm, and 17.5 kg/m^2^, respectively. Prevalence of underweight was 28.8%, and stunting was observed in 30.4%. Thinness and obesity using BMI were observed in 58.4% and 4.2%, respectively. The median body fat% was 22.5, and excess body fat (>35%) was observed in 5.7%. The prevalence of prediabetes was 39.4%. Fasting insulin concentrations, HOMA-IR, and HOMA-β showed a positive trend across body composition quartiles (*p* < 0.001). HOMA-S and compensatory β-cell response showed an inverse trend (*p* < 0.001). Compared with prediabetic girls in the overweight/obese group, girls most undernourished group had lower median insulin concentrations (8.1 μIU/ml vs. 17.1 μIU/ml), lower HOMA-IR (1.1 vs. 2.3), and lower HOMA-β (75.6 vs. 129.2) but higher sensitivity (87.4 vs. 43.7) (*p* < 0.001) for all.

**Conclusion:**

We have reported a high prevalence of prediabetes among rural adolescent girls with a very low prevalence of obesity. Prediabetes in obesity is driven by hyperinsulinemia and overworking of the pancreas while poor β-cell function and poor insulin secretion are major drivers in the undernourished group. The high-risk diabetes screening programs are much needed for the undernourished populations. Caution should be exercised for planning the interventions as overfeeding (or overnutrition) is likely to put the populations at risk of development of obesity and insulin resistance.

## Introduction

Diabetes is a global public health problem with an evident burden in developing countries such as India (1). This phenomenon is predominantly seen in urban India which has witnessed rapid nutrition and lifestyle transition over the last three decades. Prediabetes (PD) is an intermediate state of hyperglycemia with glucose levels above normal but below the diabetic threshold. There are also some recent studies from India showing an increase in not only diabetes but also PD among adolescents (2, 3). In a global report of 16 studies, the prevalence of PD ranged from 3.3% to 14.3% (4). In a meta-analysis of 52 studies analyzing comorbidities among children aged 5 to18 years, the prevalence of PD was 1.4 times higher in overweight/obese than in healthy individuals (5). There are some reports (6, 7) showing that the prevalence of type 2 diabetes (T2D) among children and adolescents is increasing in some ethnic groups. In a recent report from the US, approximately one of five adolescents and one of four adults have PD (8). Obesity is the main driving force in the development of T2D in the developed world. The old-fashioned synergy between lifestyle, obesity, and diabetes risk now needs to be revisited as there is an increasing prevalence of diabetes in undernourished populations from developing countries (9, 10). There have been recent attempts to study the pathophysiology of T2D in undernourished populations. In low- and middle-income countries (LMICs), the onset of diabetes occurs at a much earlier age and in those with low body mass index (BMI) (11). Recent reviews have identified the role of defective insulin secretion in the pathophysiology as opposed to peripheral insulin resistance observed in classical diabetes (12, 13). A longitudinal cohort study in undernourished rural adolescents found elevated glucose levels in early childhood among young adults diagnosed with glucose intolerance (14).

Undernutrition has been prevalent for many generations in the KONKAN region of the western Indian state of Maharashtra (15), but at the same time, non-communicable diseases (NCD) are on the rise (16). DERVAN cohort is a prospective cohort study of rural adolescent girls of 16–18 years old in the KONKAN region exploring the association among physical growth, nutrition, cognition in adolescence and the risk of developing NCDs (in particular diabetes) in the mothers and their offspring. The detailed protocol of the cohort study has been described earlier (17). This manuscript discusses glycemic parameters among adolescent girls at the recruitment stage of the DERVAN cohort.

## Methods

### Laboratory

Adolescent girls were brought to the institute for residential camp in groups of five to seven to ensure overnight fasting. Many of them were also accompanied by their parents. Fasting and 2-h postprandial blood samples were obtained from the girls the next morning. Random blood samples were obtained from the accompanying parent. HbA_1_C was measured using high-performance liquid chromatography (Bio-Rad D10; Bio-Rad Laboratories, Hercules, CA) calibrated against the National Glycosylated Standardization Program with a coefficient of variation (CV) of 2.8%. Blood samples were centrifuged (4°C, 3,000 rpm for 15 min) within 1 h of collection and stored at −80°C for further investigations. Blood glucose was measured on ERBA 200 with the intra- and interbatch CVs of <5%. Fasting insulin was measured on Abbott Architect i1000SR with a CV of 2.0%.

### Anthropometry and body composition

Height, weight, and circumferences (head, mid-upper arm, waist and hip) were measured using standardized protocols. Body composition was measured using a bioimpedance MC-780 analyzer (TANITA Corporation, Japan).

### Definitions

Impaired fasting glucose (IFG) was defined as fasting glucose of ≥100 mg/dl and <126 mg/dl. Elevated HbA_1_C was defined as HbA_1_C of ≥5.7% and <6.5%. We used American Diabetic Association (ADA) (18) criteria for defining PD i.e. either IFG or elevated HbA_1_C. Individuals with fasting glucose of ≥126 mg/dl or HbA_1_C of ≥6.5% were classified as diabetic (18). Normal glucose tolerance (NGT) was defined as fasting glucose of <100 mg/dl. Postprandial hyperglycemia refers to postprandial glucose of ≥140 mg/dl. Insulin resistance (HOMA-IR), insulin sensitivity (HOMA-S) and β–cell function (HOMA-β) were calculated using the homeostasis model assessment (19). We estimated compensatory β-cell response by calculating (HOMA-S) ^*^ (HOMA-β)/100 (14). Underweight and stunting were defined using the World Health Organization (WHO) growth charts for weight and height (20). Thinness and obesity were defined by the criteria laid down by the International Obesity Task Force (IOTF) (21). Adiposity was defined as body fat% of ≥35 (14) and central obesity using age-specific standard deviation (SD) score cutoff of 0.55, which corresponds to the 70^th^ percentile for waist circumference (22). Parents with no history of diabetes but random blood glucose of ≥200 mg/dl were classified as individuals with T2D.

### Statistical methods

Each variable was tested for normality using the Kolmogorov–Smirnov test. Those found not normal were appropriately transformed. The data are represented by median (25^th^-75^th^ percentile). Categorical variables are represented by frequency and percentage. For statistical analysis, body composition parameters (BMI, body fat% and waist circumference) were treated as exposures. Glycemic parameters, indices and PD were treated as outcomes. We created quartiles of exposures. We further divided fourth quartile of each exposure into two groups (non-obese and overweight/obese for BMI, non-adipose and adipose for body fat% and normal and centrally obese for waist circumference) thus eventually creating the 5 groups of each of the exposures. Group 1 and group 5 represented lower and higher extremes of each of the exposure. Anthropometry, glycemic parameters, HOMA indices and PD prevalence were compared across the five groups of each exposure using linear regression or logistic regression as appropriate. Interaction (2 way and 3 way) between body composition exposures (BMI, body fat%, and waist circumference) for outcomes (fasting glucose/PD) were tested by including appropriate product terms in linear or logistic regression. Comparison between groups was performed using the *t*-test/ANOVA/Kruskal–Wallis test/chi-square test as appropriate. Statistical software SPSS V25.0 and STATA 13.0 were used for the analysis.

### Ethics

The study was approved by the Institute Ethics Committee of BKL Walawalkar Rural Medical College and Hospital. Our institute ethics committee (EC/755/INST/MH/2015/RR-18) is registered with the Department of Health Research (DHR) Government of India. Appropriate written informed consent was obtained from the adolescent girls who were 18 years old at the time of recruitment. For those below 18 years of age, written informed consent was obtained from the parents and written informed assent was obtained from the adolescent girl. Additional consent was also obtained from parents for their own blood and clinical investigations.

## Results

We enrolled 1,520 adolescent girls between June 2019 and February 2023. Body composition measurements were taken for 1,400 subjects. Those diagnosed with diabetes (*n* = 5) were excluded. Thus, our analysis was performed on a sample of 1,395 girls.

The basic characteristics (anthropometry, body composition, glycemia and family history of T2D) of the adolescent girls are shown in Table 1.

**Table 1:** Anthropometry, body composition, glycemia and family history in adolescent girls (*n* = 1,395).

	**Parameters**	**Median (25^th^-75^th^ percentile) or *n* (%)**
Anthropometry	Age (years)	16.6 (15.8–17.3)
	Weight (kg)	40.7 (36.7–45.9)
	Underweight^$^	402 (28.8)
	Standing height (cm)	151.7 (148.2–155.6)
	Stunted^$^	424 (30.4)
	BMI (kg/m^2^)	17.5 (16.0–19.8)
	Thinness (<−1 SD) ^@^	814 (58. 4)
	Normal^@^	522 (37.4)
	Overweight/obese (> +1 SD)^@^	59 (4.2)
	Waist circumference (cm)	62.1 (58.5–67.1)
	Hip circumference (cm)	83.2 (79.6–87.8)
	WHR	0.751 (0.723–0.779)
	Central obesity^+^	24 (1.7)
Body composition	Lean mass (kg)	29.5 (27.4–31.6)
	Fat mass (kg)	8.8 (6.7–12.3)
	Body fat%	22.5 (18.6–27.6)
	Body fat% > 35	80 (5.7)
Glycemia	Serum fasting glucose (mg/dl)	95.4 (88.9–101.6)
	≥100 mg/dl and < 126 mg/dl	432 (31.0)
	Postprandial glucose (mg/dl) (*n* = 1,226)	102.0 (93.0–112.0)
	≥140 mg/dl	15 (1.2)
	HBA_1_C (%)	5.3 (5.0–5.5)
	≥5.7% and < 6.4%	192 (13.8)
	Prediabetes	550 (39.4)
	Insulin (μIU/ml)	8.6 (6.9–11.1)
	HOMA-IR	1.2 (0.9–1.5)
	HOMA-β	94.5 (79.4–113.6)
	HOMA-S	86.1 (66.6–108.1)
	Compensatory β-cell response	79.6 (67.2–97.5)
Family history of T2D	Father (*n* = 144)	Known: 7 Newly diagnosed:4
	Mother (*n* = 507)	Known:14 Newly diagnosed:5

^$^As per WHO (20); ^@^as per IOTF (International Obesity Task Force) (21); WHR: waist circumference-to-hip circumference ratio; ^+^Indian Academy of Pediatrics (22); T2D: type 2 diabetes; SD: standard deviation.

### Anthropometry

The median age at enrollment was 16.6 years. The median weight, height and BMI were 40.7 kg, 151.7 cm and 17.5 kg/m^2^ respectively. Using the WHO classification, 28.8% were classified as underweight and 30.4% were stunted. Thinness and overweight/obese using the IOTF standards were observed in 58.4% and 4.2% respectively. The median waist circumference, hip circumference and waist-to-hip ratio were 62.1 cm, 83.2 cm and 0.751 respectively. Only 1.7% of the girls were centrally obese.

### Body composition

The median lean mass and fat mass were 29.5 kg and 8.8 kg respectively. The median body fat% was 22.5 and 5.7% of the girls had body fat% of >35.

### Glycemia

The median fasting glucose, HbA_1_C and insulin concentrations were 95.4 mg/dl, 5.3% and 8.6 μIU/ml respectively. IFG was diagnosed in 31.0% and elevated HbA_1_C in 13.8%. A total of 39.4% of the girls had PD. The median values for various glycemic indices calculated using the homeostasis model were 1.2 for HOMA-IR, 94.5 for HOMA-β, 86.1 for HOMA-S and 79.6 for compensatory β-cell response. We were able to collect postprandial blood samples only in 1,226 girls. Median postprandial glucose concentration was 102.0 mg/dl and only 15 (1.2%) had postprandial hyperglycemia. Out of these 11 were IFG.

### Family history

Parents (144 fathers, 507 mothers) of 547 adolescent girls reported to the institute for blood investigations; 7 fathers and 14 mothers were known diabetic and 4 fathers and 5 mothers were newly diagnosed with diabetes.

#### Anthropometry and body composition between PD and NGT

Table 2 shows a comparison of anthropometric and body composition parameters among girls with PD and NGT. BMI and waist circumference were similar. Waist-to-hip ratio was significantly higher in those with PD (*p* = 0.041). All the remaining parameters were similar.

**Table 2:** Anthropometry and body composition between girls with PD and NGT (*n* = 1,395).

**Anthropometry**	**PD (*n* = 550)**	**NGT (*n* = 845)**	***P*-value**
Age (years)	16.6 (15.8–17.3)	16.6 (15.8–17.3)	0.602
Weight (kg)	40.7 (36.7–46.1)	40.7 (36.7–45.7)	0.661
Underweight^$^	158 (28.7)	244 (28.9)	0.952
Standing height (cm)	151.7 (148.3–155.6)	151.7 (148.2–155.6)	0.647
Stunted^$^	164 (29.8)	260 (30.8)	0.706
BMI (kg/m^2^)	17.6 (16.0–19.8)	17.5 (16.0–19.8)	0.552
Thinness (< −1 SD) ^@^	314 (57.1)	500 (59.2)	
Normal^@^	208 (37.8)	314 (37.2)	0.391
Overweight/obese (> +1 SD) ^@^	28 (5.1)	31 (3.7)	
Waist circumference (cm)	62.3 (58.3–67.6)	62.0 (58.5–66.7)	0.264
Hip circumference (cm)	83.2 (79.5–88.0)	83.2 (79.7–87.6)	0.972
WHR	0.752 (0.727–0.780)	0.750 (0.721–0.777)	0.041^*^
**Body composition**			
Lean mass (kg)	29.6 (27.4–31.8)	29.3 (27.4–31.6)	0.388
Body fat mass (kg)	8.7 (6.7–12.3)	8.9 (6.8–12.2)	0.766
Body fat%	22.4 (18.4–27.5)	22.6 (18.7–27.7)	0.964

Median (25^th^-75^th^ percentile) or n (%); ^*^ statistically significant (*p* < 0.05) by the Kruskal–Wallis test for continuous data and chi-square test for categorical data; ^$^as per WHO (20); ^@^as per IOTF (International Obesity Task Force) (21); WHR: waist circumference-to-hip circumference ratio; ^+^Indian Academy of Pediatrics (22); SD: standard deviation.

#### Anthropometry, body composition and glycemic parameters

Table 3 shows anthropometric and glycemic parameters across five BMI groups. Anthropometric and body composition parameters except height showed a positive trend across the BMI groups. Fasting glucose concentrations, HbA_1_C and the proportion of those with PD were similar across the five groups. Fasting insulin concentrations, HOMA-IR and HOMA-β showed a positive trend across the BMI groups (*p* < 0.001 for all). HOMA-S and compensatory β-cell response showed an inverse trend (*p* < 0.001 for both). Similar results were obtained when we used other exposures such as body fat% (Supplementary Table 1) and waist circumference (Supplementary Table 2).

**Table 3:** Anthropometry and glycemia according to BMI (*n* = 1,395).

**Minimum–maximum**	**Q1 (*n* = 348) (12.2 kg/m^2^-16.0 kg/m^2^)**	**Q2 (*n* = 349) (16.0 kg/m^2^-17.5 kg/m^2^)**	**Q3 (*n* = 349) (17.5 kg/m^2^-19.8 kg/m^2^)**	**Q4 (Normal BMI) (*n* = 290) (19.8 kg/m^2^-24.6 kg/m^2^)**	**Q4 (Ovwt/obese) (*n* = 59) (25.0 kg/m^2^-38.3 kg/m^2^)**	***p***
Weight (kg)	34.6 (32.7–36.9)	38.5 (36.8–40.9)	42.4 (40.5–44.9)	49.0 (46.6–52.3)	63.3 (59.0–70.8)	0.000^*^
Height (cm)	152.0 (148.1–156.0)	152.0 (148.3–155.8)	151.5 (148.5–155.0)	151.7 (147.9–155.4)	152.1 (148.4–156.8)	0.374
WHR	0.734 (0.707–0.758)	0.741 (0.717–0.768)	0.753 (0.728–0.776)	0.773 (0.745–0.813)	0.805 (0.780–0.841)	0.000^*^
Fat mass (kg)	5.7 (4.9–6.8)	7.7 (6.8–8.7)	10.0 (8.8–11.4)	14.4 (12.9–16.4)	24.8 (21.9–30.4)	0.000^*^
Lean mass (kg)	27.0 (25.3–28.4)	28.6 (27.2–30.5)	30.3 (28.6–31.7)	32.0 (30.5–33.6)	35.1 (33.4–38.0)	0.000^*^
Fasting glucose (mg/dl)	94.7 (88.7–100.7)	95.2 (88.6–101.2)	95.6 (88.9–102.0)	96.0 (90.1–102.1)	97.2 (85.3–102.0)	0.738
HBA_1_C (%)	5.3 (5.0–5.5)	5.2 (5.0–5.5)	5.2 (5.0–5.5)	5.3 (5.0–5.5)	5.4 (5.1–5.6)	0.363
Prediabetes n (%)	135(38.8)	134(38.4)	144(41.3)	109(37.6)	28(47.5)	0.607
Fasting insulin (μIU/mL)	7.6 (5.9–9.6)	8.0 (6.4–10.3)	8.8 (7.1–10.8)	10.1 (8.3–12.4)	14.7 (10.9–17.6)	0.000^*^
HOMA-IR	1.0 (0.8–1.3)	1.1 (0.9–1.4)	1.2 (1.0–1.5)	1.4 (1.1–1.7)	2.0 (1.5–2.4)	0.000^*^
HOMA-β	85.8 (73.2–103.2)	90.7 (76.6–108.2)	94.2 (80.2–111.7)	104.9 (89.0–124.1)	134.0 (109.0–161.6)	0.000^*^
HOMA-S	97.7 (77.8–125.1)	92.5 (71.5–115.7)	83.4 (68.2–104.9)	73.4 (58.3–89.5)	50.4 (41.4–67.1)	0.000^*^
Compensatory β-cell response	84.4 (70.8–102.7)	81.7 (69.1–100.3)	79.2 (67.1–96.4)	74.8 (64.4–89.9)	67.5 (55.1–91.2)	0.000^*^

Median (25^th^-75^th^ percentile) or *n* (%); ^*^ statistically significant (*p* < 0.05) by the Kruskal–Wallis test for continuous data and the chi-square test for categorical data; Q1, Q2, Q3, and Q4 are quartiles of BMI. Q4 is further divided into two groups of normal BMI and those overweight/obese; Ovwt: overweight.

**Figure 1:**
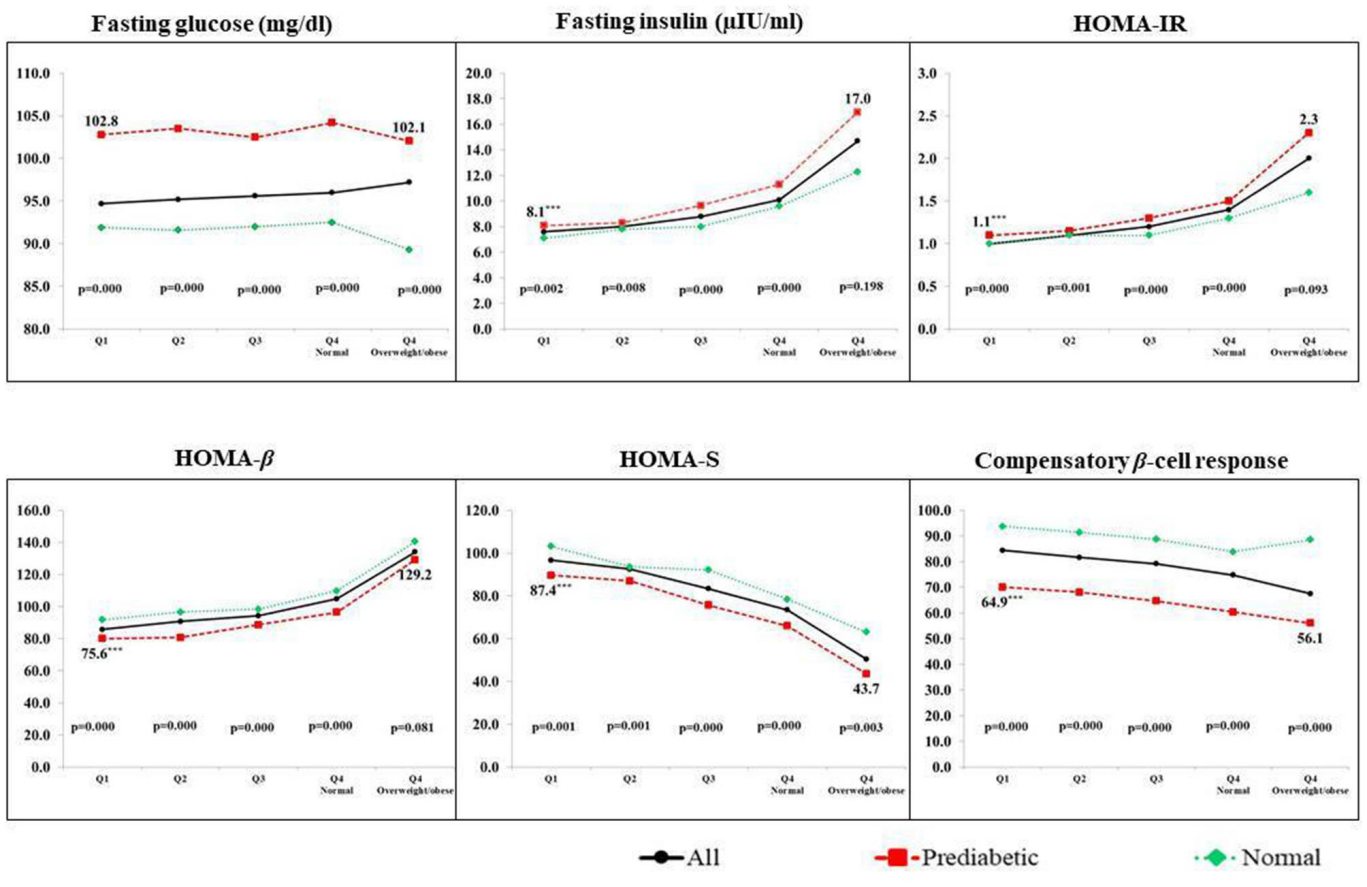
Glycemic parameters of PD and NGT adolescent girls across the BMI quartiles. Q1, Q2, Q3, and Q4 are quartiles of BMI. Q4 is further divided into two groups of normal BMI and those overweight/obese; *p*-value by the Kruskal–Wallis test. Data are represented by median values.

We also compared glycemic parameters between girls with PD and NGT in each of the five groups of BMI (Figure 1). In each group, those diagnosed with PD had significantly lower HOMA-β, lower HOMA-S, and lower compensatory β-cell response but higher HOMA-IR and higher insulin concentrations. This was also true when we used other exposures such as body fat% (Supplementary Figure 1) and waist circumference (Supplementary Figure 2).

#### Glycemia among undernourished and overnourished prediabetics

We also compared glycemic parameters among girls with PD in the two extremes of the exposures. In the case of BMI, girls with PD in the most undernourished group (Q1) when compared to girls with PD in the overweight/obese group had significantly lower insulin concentrations (8.1 μIU/ml vs. 17.0 μIU/ml), lower insulin resistance (1.1 vs. 2.3), lower β-cell function (75.6 vs. 129.2) but higher sensitivity (87.4 vs. 43.7) (*p* < 0.001 for all) (Figure 1). Similar results were obtained when we compared girls with PD in extreme groups of other exposures such as body fat% (Supplementary Figure 1) and waist circumference (Supplementary Figure 2).

#### Interaction between body composition parameters for fasting glucose/PD

Using continuous data, there was no statistically significant 2-way interaction between BMI and body fat%, BMI and waist circumference and body fat% and waist circumference for fasting glucose concentrations. The three-way interaction between BMI, body fat% and waist circumference was also not significant. Using a categorical approach, there were no statistically significant interactions (2 way or 3 way) for PD.

## Discussion

We have described glycemic parameters in rural adolescent girls aged 16 to18 years in the KONKAN region. Prevalence of obesity (using BMI), adiposity (using body fat%) and central obesity (using waist circumference) was very low. The proportion of girls with PD was 39.4% and postprandial hyperglycemia was observed only in 1.2%. Prediabetes has been characterized by the simultaneous presence of insulin resistance and β-cell dysfunction abnormalities that start before glucose changes are detectable (23). According to DeFronzo et al. (24) impaired glucose tolerance (IGT) is characterized by severe muscle insulin resistance with mild hepatic insulin resistance, while IFGs are by severe hepatic insulin resistance with normal or near-normal muscle insulin sensitivity. The possible explanation for fasting hyperglycemia in our study could be due to compensatory hepatic efflux as suggested by Turner et al. (25). There are many reports about PD among Indian adolescents with wide variations in prevalence estimates. A study on 185 hostel students from the northern Indian city of Kanpur reported a PD prevalence of 32.5% (26). A prevalence of 6.8% was reported in a small study from a rural area of the southern Indian state of Tamil Nadu (27). Another study from the same state reported an increased prevalence of PD at follow-up (28). There were also three reports from the city of Chennai. A study among overweight and obese adolescents reported a prevalence of 11.7% (29). Another study among 1,519 children (6–11 years old) and (12–19 years old) found a prevalence of 3.7%, but it was very high at 12.7% in girls with abdominal obesity. Waist circumference and BMI were the main determinants (3). A longitudinal study among adolescents reported an increase in the incidence of PD from 3.7% at baseline to 13.6% at a median follow-up of 7 years (30). A study in another southern Indian state of Karnataka among children and adolescents aged 6–18 years admitted at tertiary care hospitals reported a PD prevalence of 20.4% (31). A cohort study from India has also reported a high prevalence of 20% in 18 year old adolescent girls (14). Recently concluded Comprehensive National Nutrition Survey (2) found a prevalence of 8.4% among adolescent girls where an HbA_1_C of >5.8% cutoff was used for diagnosis. We used ADA criteria as it has been shown to improve the parity between IFG and IGT (32).

Girls in our cohort are undernourished, yet have a high prevalence of PD. In most of the studies on PD in adolescents, the subjects were either overweight or obese. Our study is being carried out in a region witnessing undernutrition from birth to adolescence (15) as well as in adulthood (33). In our data, we have observed an increasing trend for HOMA-IR and β-cell function and a decreasing trend for insulin sensitivity across varying BMI, body fat% and waist circumference groups. The major pathophysiology for T2D in undernourished people is impaired pancreatic insulin secretion and rapid β-cell failure as against insulin resistance observed in obese people (10, 13). Poor insulin secretion has been ascribed to poor β-cell mass as suggested by the concept of Developmental Origins of Health and Disease (DOHAD) by David Barker where children who have been exposed to undernutrition during fetal life and in the early postnatal period develop a risk of T2D in adulthood (34). Thus, PD in our girls might have early life origins. When we compared girls with PD and NGT in each BMI group, girls with PD had significantly lower β-cell function and insulin sensitivity. In our study, we found the lowest insulin concentration in those with low BMI. Recently, a study by Yajnik et al. (35) from India identified a subgroup of individuals with severely insulin-deficient diabetes (SIDD), which was characterized by early onset, low insulin secretion, relatively low BMI and poor metabolic control. There is also another report from India (10) where insulin secretory capacity in lean/low BMI adult individuals with diabetes was lowest when compared with individuals with T2D or controls but higher than those with type 1 diabetes. None of the girls in our analysis are diabetic but the high prevalence of PD coupled with similar findings found in the above reports (10, 35) indicates that these girls are already at risk of developing T2D unlike classical T2D driven by insulin resistance.

β-cell secretory capacity and age-related insulin insensitivity were identified as causes of glucose intolerance among young adults in a longitudinal cohort study (14). Our study is also longitudinal but has a cross-sectional nature at the first stage of cohort recruitment. We also found similar results (decreasing β-cell response and insulin sensitivity) in our girls with PD across all the BMI groups. The prevalence of overweight/obesity (4.2%) as well as central obesity (1.7%) was very low. We used the quartile approach for balanced statistical comparisons. As expected, we observed higher insulin resistance, lower β-cell function and hyperinsulinemia in overweight/obese girls indicating overload on the pancreas but overweight/obese girls with PD had higher insulin resistance with higher β-cell function and low insulin sensitivity when compared with their NGT counterparts. This indicated failure of compensatory mechanisms which is usually seen in obese people.

Studies report a conversion rate from PD to diabetes of ~5–10%, which varies in different populations and by definition of PD (36–38). Several lifestyle and drug intervention trials among prediabetic adults have shown a reduction in subsequent diabetes risk (38, 39). Prediabetes or diabetes in overweight/obese people is driven by increased insulin resistance. Weight loss and lifestyle modifications have shown reversal to normoglycemia in overweight/obese populations. But similar strategies in undernourished populations might aggravate the situation leading to bone loss and sarcopenic obesity (13). Therefore, strategies to contain diabetes among undernourished populations should focus on the restoration of insulin sensitivity as we cannot improve insulin secretion due to fetal programming. In addition to this, insulin sensitivity is known to decrease in pregnancy. An appropriate drug or nutritional intervention will be beneficial for the prevention of T2D as well as gestational diabetes (GDM) in the current generation and their offspring. Micronutrients are known to play a role in glucose metabolism and insulin signaling (40, 41). The nutritional intervention strategies should focus on ensuring not only adequate macronutrients but also micronutrients.

Data were collected at the recruitment stage of the cohort, where measurement of glucose was taken only once. Only when we have glucose measurements at subsequent follow-ups, we will be able to study the evolution of glucose tolerance.

There are some limitations in our study. We did not do the oral glucose tolerance test (OGTT); hence, we could not assess IGT and first-phase insulin secretion in our girls. Our study sample consists only of girls; hence, we cannot generalize our findings.

## Conclusion

To summarize, the subjects in our analysis sample are younger and non-diabetic but still have a high prevalence of PD with poor insulin levels. High-risk screening programs for diabetes should also concentrate on the undernourished populations. Caution should be exercised for planning the interventions to restore insulin sensitivity. Overfeeding only macronutrients is likely to put the populations at risk of developing insulin resistance leading to T2D in future. Only systematic follow-up of our cohort will tell us about the causality of diabetes in the undernourished.

## Data availability statement

The datasets presented in this article are not readily available because the datasets generated are available from the corresponding author on reasonable request with necessary permissions from Institutional Ethics Committee and Government of India HMSC permission. Requests to access the datasets should be directed to SP, dr.suvrnanpatil@gmail.com.

## Ethics statement

The studies involving human participants were reviewed and approved by our Institute Ethics Committee (EC/755/INST/MH/2015/RR-18) is registered with the Department of Health Research (DHR) Government of India. Written informed consent to participate in this study was provided by the participants' legal guardian/next of kin.

## Author contributions

SP is the principal investigator of the study and wrote the first draft and discussion section. NP and PH-B helped in literature review, writing of the manuscript, and carried out laboratory assays. OD managed and analyzed the data. ANa carried out laboratory assays. AY and ANi also helped in the interpretation of laboratory data. CJ carried out literature review, results interpretation, writing of manuscript, and also helped in discussion. RB participated in laboratory assays and final editing. All authors contributed to the article and approved the submitted version.

## References

[1] Sun H Saeedi P Karuranga S Pinkepank M Ogurtsova K Duncan BB. IDF diabetes Atlas: global, regional and country-level diabetes prevalence estimates for 2021 and projections for 2045. Diabetes Res Clin Pract. (2022) 183:109119. 10.1016/j.diabres.2021.109119 34879977 PMC11057359

[2] Kumar P Srivastava S Mishra PS Mooss ETK. Prevalence of pre-diabetes/type 2 diabetes among adolescents (10–19 years) and its association with different measures of overweight/obesity in India: a gendered perspective. BMC Endocr Disord. (2021) 21:146. 10.1186/s12902-021-00802-w 34233661 PMC8261995

[3] Ranjani H Sonya J Anjana RM Mohan MV. Prevalence of glucose intolerance among children and adolescents in urban South India (ORANGE-2). Diabetes Technol Ther. (2013) 15:13–9. 10.1089/dia.2012.0236 23151017

[4] Spurr S Bally J Hill P Gray K Newman P Hutton A. Exploring the prevalence of undiagnosed prediabetes, type 2 diabetes mellitus, and risk factors in adolescents: a systematic review. J Pediatr Nurs. (2020) 50:94–104. 10.1016/j.pedn.2019.09.025 31786470

[5] Sharma V Coleman S Nixon J Sharples L Hamilton-Shield J Rutter H. A systematic review and meta-analysis estimating the population prevalence of comorbidities in children and adolescents aged 5 to 18 years. Obes Rev. (2019) 20:1341–9. 10.1111/obr.12904 31342672 PMC6851579

[6] Runge CR Ng M Herman WH Gebremariam A Hirschfeld E Lee JM. Racial differences in prediabetes prevalence by test type for the US pediatric and adult population: NHANES 1999-2016. Pediatr Diabetes. (2020) 21:1110–5. 10.1111/pedi.13083 32681534 PMC10771709

[7] Prediabetes/type 2 diabetes prehypertension/hypertension prehypertension/hypertension and obesity among ethnic groups of adolescents in Western Canada. BMC Pediatr. (2020) 20:31. 10.1186/s12887-020-1924-6 31973728 PMC6979336

[8] Andes LJ Cheng YJ Rolka DB Gregg EW Imperatore G. Prevalence of prediabetes among adolescents and young adults in the United States, 2005–2016. JAMA Pediatr. (2020) 174:e194498. 10.1001/jamapediatrics.2019.4498 31790544 PMC6902249

[9] Maiti S Sinha NK Khan MM Das PK Chattopadhyay JC. Diabetes in rural individuals of different nutritional status and the alarming situation demands focus more on its under-nutrition association. Arch Physiol Biochem. (2015) 121:26–31. 10.3109/13813455.2014.959973 25244251

[10] Lontchi-Yimagou E Dasgupta R Anoop S Kehlenbrink S Koppaka S Goyal A. An atypical form of diabetes among individuals with low BMI. Diabetes Care. (2022) 45:1428–37. 10.2337/dc21-1957 35522035 PMC9184261

[11] Yajnik CS. Early life origins of insulin resistance and type 2 diabetes in India and other Asian countries. J Nutr. (2004) 134:205–10. 10.1093/jn/134.1.205 14704320

[12] Ahmed S PrayGod G R Lee N Kelly P Trilok-Kumar G Chisenga M. Long-term health after severe acute malnutrition in children and adults- the role of the pancreas (SAMPA): protocol. F1000Res. (2022) 11:777. 10.12688/f1000research.123389.2 36300035 PMC9577280

[13] George AM Jacob AG Fogelfeld L. Lean diabetes mellitus: an emerging entity in the era of obesity. World J Diabetes. (2015) 6:613–20. 10.4239/wjd.v6.i4.613 25987958 PMC4434081

[14] Yajnik CS Bandopadhyay S Bhalerao A Bhat DS Phatak SB Wagh RH. Poor in utero growth, and reduced β-cell compensation and high fasting glucose from childhood, are harbingers of glucose intolerance in young Indians. Diabetes Care. (2021) 44:2747–57. 10.2337/dc20-3026 34610922

[15] Patil S Joglekar C Chavan R Sonawane S Modak A. Trends in malnutrition indicators from birth to adolescence in rural KOKAN Region of Western India. Int J Nutr Sci. (2020) 5:1041.

[16] Kshirsagar MV Ashturkar MD. Prevalence of lifestyle diseases in Maharashtra: a comparison between NFHS-5 and NFHS-4 surveys. J Family Med Prim Care. (2022) 11:2474–8. 10.4103/jfmpc.jfmpc_1944_21 36119353 PMC9480665

[17] Patil S Patil N Joglekar C Yadav A Nilawar A Banavali U. An adolescent and preconception health perspective of adult non communicable diseases (DERVAN): Protocol for rural prospective adolescent girls cohort study in Ratnagiri district of Kokan region of India (DERVAN-1). BMJ Open. (2020) 10:e035926. 10.1136/bmjopen-2019-035926 32895267 PMC7476477

[18] Classification and Diagnosis of Diabetes. Standards of medical care in diabetes-2020. Diabetes Care. (2020) S14–31. 10.2337/dc20-S002 31862745

[19] iHOMA2. Available online at: https://www.phc.ox.ac.uk/research/technology-outputs/ihoma2 (accessed December 24, 2022).

[20] The WHO Child Growth Standards. Available online at: http://www.who.int/childgrowth/standards/en/ (accessed March 12, 2023).

[21] Cole T Lobstein T. Extended international (IOTF) body mass index cut-offs for thinness, overweight and obesity. Pediatric Obes. (2012) 7:284–94. 10.1111/j.2047-6310.2012.00064.x 22715120

[22] Khadilkar A Ekbote V Chiplonkar S Khadilkar V Kajale N Kulkarni S. Waist circumference percentiles in 2–18-year-old Indian children. J Pediatr. (2014) 164:1358–62.e2. 10.1016/j.jpeds.2014.02.018 24655536

[23] Tabák AG Herder C Rathmann W Brunner EJ Kivimäki M. Prediabetes: a high-risk state for diabetes development. Lancet. (2012) 379:2279–90. 10.1016/S0140-6736(12)60283-9 22683128 PMC3891203

[24] Abdul-Ghani MA Tripathy D DeFronzo RA. Contributions of beta-cell dysfunction and insulin resistance to the pathogenesis of impaired glucose tolerance and impaired fasting glucose. Diabetes Care. (2006) 29:1130–9. 10.2337/dc05-2179 16644654

[25] Turner RC Holman RR Matthews D Hockaday TD Peto J. Insulin deficiency and insulin resistance interaction in diabetes: estimation of their relative contribution by feedback analysis from basal plasma insulin and glucose concentrations. Metabolism. (1979) 28:1086–96. 10.1016/0026-0495(79)90146-X 386029

[26] Midha T Krishna V Shukla R Katiyar P Kaur S Martolia DS. Correlation between hypertension and hyperglycemia among young adults in India. World J Clin Cases. (2015) 3:171–9. 10.12998/wjcc.v3.i2.171 25685764 PMC4317611

[27] Taranikanti M Panda S Sukanya M Swamy PN Khan MS Tabassum H. Prediabetes in South Indian rural adolescent school students. Indian J Physiol Pharmacol. (2014) 58:77–80. 25464681

[28] Nanditha A Snehalatha C Satheesh K Susairaj P Simon M Vijaya L. Secular trends in diabetes in India (STRiDE-I): change in prevalence in 10 years among urban and rural populations in Tamil Nadu. Diabetes Care. (2019) 42:476–85. 10.2337/dc18-1559 30659076

[29] Krishnappa SK Yashoda HT Boraiah G Vishwa S. Sagittal abdominal diameter to measure visceral adipose tissue in overweight or obese adolescent children and its role as a marker of insulin resistance. J Clin Diagn Res. (2015) 9:SC09–12. 10.7860/JCDR/2015/15971.6742 26673888 PMC4668495

[30] Mehreen TS Kamalesh R Pandiyan D Kumar DS Anjana RM Mohan V. Incidence and predictors of dysglycemia and regression to normoglycemia in indian adolescents and young adults: 10-year follow-up of the orange study. Diabetes Technol Ther. (2020) 22:875–82. 10.1089/dia.2020.0109 32349530

[31] Aroor AR Vaghese TC Soans ST. Prevalence of prediabetes in children and its association with risk factors. Int J Contemp Pediatr. (2019) 6:1957–63. 10.18203/2349-3291.ijcp20193705

[32] Hostalek U. Global epidemiology of prediabetes - present and future perspectives. Clin Diabetes Endocrinol. (2019) 5:5. 10.1186/s40842-019-0080-0 31086677 PMC6507173

[33] Patil SN. Ostensibly healthy forerunners of non-communicable diseases (NCD) from rural KONKAN, India. CODH-V7. (2023) 3:107–16. 10.9734/bpi/codh/v7/3698B

[34] Hales CN Barker DJ. Type 2 (non-insulin-dependent) diabetes mellitus: the thrifty phenotype hypothesis. Diabetologia. (1992) 35:595–601. 10.1007/BF00400248 1644236

[35] Yajnik CS Wagh R Kunte P Asplund O Ahlqvist E Bhat DS. Polygenic scores of diabetes-related traits in subgroups of type 2 diabetes in India: a cohort study. The Lancet Regional Health - Southeast Asia. (2023) 1–14. 10.1016/j.lansea.2023.100182 37492423 PMC10363502

[36] Forouhi NG Luan J Hennings S Wareham NJ. Incidence of Type 2 diabetes in England and its association with baseline impaired fasting glucose: the Ely study 1990–2000. Diabet Med. (2007) 24:200–7. 10.1111/j.1464-5491.2007.02068.x 17257284

[37] Nathan DM Davidson MB DeFronzo RA Heine RJ Henry RR Pratley R. American Diabetes Association. Impaired fasting glucose and impaired glucose tolerance: implications for care. Diabetes Care. (2007) 30:753–9. 10.2337/dc07-9920 17327355

[38] Diabetes Prevention Program Research Group Knowler WC Fowler SE Hamman RF Christophi CA Hoffman HJ. 10-year follow-up of diabetes incidence and weight loss in the diabetes prevention program outcomes study. Lancet. (2009) 374:1677–86. 10.1016/S0140-6736(09)61457-4 19878986 PMC3135022

[39] Ramachandran A Snehalatha C Mary S Mukesh B Bhaskar AD Vijay V. Indian Diabetes Prevention Programme (IDPP). The Indian Diabetes Prevention Programme shows that lifestyle modification and metformin prevent type 2 diabetes in Asian Indian subjects with impaired glucose tolerance (IDPP-1). Diabetologia. (2006) 49:289–97. 10.1007/s00125-005-0097-z 16391903

[40] Via M. The malnutrition of obesity: micronutrient deficiencies that promote diabetes. ISRN Endocrinol. (2012) 2012:103472. 10.5402/2012/103472 22462011 PMC3313629

[41] Zhang D Zhong X Cheng C Su Z Xue Y Liu Y. Effect of vitamin D and/or calcium supplementation on pancreatic β-cell function in subjects with prediabetes: a randomized, controlled trial. J Agric Food Chem. (2023) 71:347–57. 10.1021/acs.jafc.2c05469 36541437

