# Supplementary material for: Prediabetes in rural adolescent girls from DERVAN cohort: data from the KONKAN region of the state of Maharashtra, India (DERVAN-4)
Source: Front Public Health. 2023 Aug 3;11:1181401. doi: 10.3389/fpubh.2023.1181401 (PMC10435731; doi:10.3389/fpubh.2023.1181401)
Supplement: Supplementary file 1 [file Data_Sheet_1.pdf]

# SUPPLEMENTARY MATERIALS

## Prediabetes in rural adolescent girls from DERVAN cohort
## Data from the KONKAN region of the state of Maharashtra, India. (DERVAN-4)

Suvarna Patil MD[1], Netaji Patil DMRE[2], Pallavi Hardikar-Bhat MSc[3], Omkar Dervankar MSc[3], Charudatta Joglekar MS[3], Rohit Bhat PhD[3], Ajit Nandoskar DMLT[3], Arvind Yadav PhD[4], Anup Nilawar MD[4]

1. Department of Medicine, BKL Walawalkar Rural Medical College, Sawarde, Taluka-Chiplun, District-Ratnagiri, Maharashtra, India
2. Department of Radiology, BKL Walawalkar Hospital and Rural Medical College, Sawarde, Taluka-Chiplun, District-Ratnagiri, Maharashtra, India
3. Regional Centre for Adolescent Health and Nutrition, BKL Walawalkar Rural Medical College, Sawarde, Taluka-Chiplun, District-Ratnagiri, Maharashtra, India
4. Department of Biochemistry, BKL Walawalkar Rural Medical College, Sawarde, Taluka-Chiplun, District-Ratnagiri, Maharashtra, India

**Supplementary Table 1: Anthropometry and glycemia according to fat % (n=1395)**

| Minimum-Maximum | Q1 (n=343) (6.1% -18.5%) | Q2 (n=351) (18.6%-22.4%) | Q3 (n=351) (22.5% - 27.5%) | Q4 (Fat% <=35) (n=270) (27.6% - 35.0%) | Q4 (Fat%>35) (n=80) (35.3% - 53.8%) | P |
|---|---|---|---|---|---|---|
| Weight (kg) | 35.2 (33.0 – 38.0) | 38.4 (36.3 – 41.4) | 41.9 (39.5 – 45.3) | 48.4 (44.8 – 51.4) | 61.1 (55.4 – 67.6) | 0.000* |
| Height (cm) | 151.5 (147.6 – 155.4) | 152.0 (148.7- 155.5) | 152.0 (148.9 – 155.9) | 151.4 (147.9 – 155.5) | 153.1 (149.2 – 156.8) | 0.264 |
| WHR | 0.736 (0.710 – 0.762) | 0.741 (0.715 – 0.768) | 0.751 (0.726 – 0.775) | 0.768 (0.743 – 0.807) | 0.806 (0.780 – 0.840) | 0.000* |
| Fat mass (kg) | 5.5 (4.7 – 6.3) | 7.8 (7.1 – 8.4) | 10.3 (9.3 – 11.4) | 14.3 (13.0 – 16.0) | 23.2 (20.1 – 28.1) | 0.000* |
| Lean mass (kg) | 28.0 (26.3 – 29.7) | 28.6 (27.1 – 30.5) | 29.6 (27.7 – 31.6) | 31.4 (29.5 – 33.1) | 34.3 (32.1 – 37.5) | 0.000* |
| Fasting glucose (mg/dl) | 95.7 (89.4 - 101.9) | 94.5 (88.6 - 100.5) | 95.1 (88.9 - 101.2) | 96.0 (89.2 - 102.1) | 97.1 (89.2 - 102.1) | 0.754 |
| $HBA_1C$ (%) | 5.3 (5.1 - 5.5) | 5.3 (5.0 - 5.5) | 5.2 (5.0 - 5.5) | 5.3 (5.0 - 5.5) | 5.3 (5.1 - 5.6) | 0.403 |
| Prediabetes n (%) | 141 (41.1) | 138 (39.3) | 135 (38.5) | 100 (37.0) | 36 (45.0) | 0.693 |
| Fasting insulin (µIU/mL) | 7.8 (6.1 - 9.7) | 7.9 (6.3 - 10.1) | 8.6 (6.9 - 11.0) | 9.9 (8.1 - 12.2) | 13.2 (10.7 - 17.2) | 0.000* |
| HOMA-IR | 1.1 (0.8 - 1.3) | 1.1 (0.9 - 1.4) | 1.2 (0.9 - 1.5) | 1.3 (1.1 - 1.7) | 1.8 (1.4 - 2.4) | 0.000* |
| HOMA-β | 85.6 (72.1 - 103.4) | 90.5 (78.0 – 106.3.) | 95.5 (81.0 - 114.2) | 103.1 (88.9 – 124.8) | 126.1 (104.8 - 156.6) | 0.000* |
| HOMA-S | 94.5 (75.9 - 118.0) | 93.4 (73.8 - 115.9) | 85.8 (67.1 - 107.0) | 75.6 (60.3 - 90.9) | 56.9 (43.0 - 71.5) | 0.000* |
| Compensatory $\beta$-cell response | 81.2 (70.1 - 100.6) | 83.2 (70.7 - 100.7) | 80.2 (65.9 - 97.0) | 75.3 (65.0 - 92.0) | 68.0 (59.9 - 89.5) | 0.000* |

**Legends for Supplementary Table 1:** Median ($25^{th}$-$75^{th}$ centile) or n (%); * statistically significant ($p<0.05$) by Kruskal-Wallis Test for continuous data and Chi square test for categorical data; Q1, Q2, Q3, Q4 are quartiles of body fat%. Q4 is further divided into 2 groups of (Fat% ≤ 35) and (Fat% >35)

**Supplementary Table 2: Anthropometry and glycemia according to waist circumference (n=1389)**

| Minimum-Maximum | Q1 (n=344) (46.8 cm - 58.4 cm) | Q2 (n=351) (58.5 cm - 62.1 cm) | Q3 (n=345) (62.1 cm - 67.0 cm) | Q4 (Normal) (n=325) (67.1 cm - 85.2 cm) | Q4 (Centrally obese) (n=24) (84.9 cm - 106.1 cm) | p |
|---|---|---|---|---|---|---|
| Weight (kg) | 34.8 (32.8 – 37.1) | 38.4 (36.7 – 40.9) | 42.2 (40.2 – 44.9) | 49.6 (45.9 – 53.6) | 71.6 (62.1 – 80.1) | 0.000 * |
| Height (cm) | 150.1 (147.2 – 154.3) | 151.4 (147.6 – 155.5) | 152.9 (149.3 – 156.5) | 152.5 (149.5 – 156.3) | 154.0 (150.2 – 158.2) | 0.000* |
| WHR | 0.717 (0.694 – 0.738) | 0.739 (0.717 – 0.760) | 0.759 (0.738 – 0.778) | 0.793 (0.766 – 0.822) | 0.851 (0.801 – 0.892) | 0.000* |
| Fat mass (kg) | 5.9 (4.9 – 7.1) | 7.9 (6.6 – 9.1) | 9.8 (8.4 – 11.4) | 14.5 (10.6 – 17.5) | 31.8 (24.7 – 37.6) | 0.000* |
| Lean mass (kg) | 27.1 (25.4 – 28.3) | 28.4 (27.0 – 30.3) | 30.3 (28.7 – 31.7) | 32.2 (30.7 – 33.9) | 37.8 (35.0 – 39.2) | 0.000* |
| Fasting glucose (mg/dl) | 95.0 (88.7 - 101.2) | 94.6 (88.1 - 100.4) | 95.0 (89.1 - 101.8) | 97.1 (90.4 - 102.9) | 98.4 (90.6 -102.3) | 0.056 |
| $HBA_1C$ (%) | 5.3 (5.0 - 5.5) | 5.2 (5.0 - 5.5) | 5.3 (5.0 - 5.5) | 5.3 (5.0 - 5.5) | 5.4 (5.2 - 5.6) | 0.270 |
| Prediabetes n (%) | 140 (40.7) | 126 (35.9) | 131 (38.0) | 141 (43.3) | 11 (45.8) | 0.297 |
| Fasting insulin (µIU/mL) | 7.6 (5.9 - 9.4) | 7.9 (6.4 -10.0) | 8.9 (7.1 - 11.0) | 10.3 (8.5 - 12.7) | 16.6 (13.7 - 18.9) | 0.000* |
| HOMA-IR | 1.0 (0.8 - 1.3) | 1.1 (0.9 - 1.4) | 1.2 (1.0 - 1.5) | 1.4 (1.1 - 1.7) | 2.3 (1.8 - 2.5) | 0.000* |
| HOMA-β | 85.6 (73.9 - 102.9) | 90.6 (76.4 - 107.1) | 97.5 (81.0 - 116.1) | 105.7 (89.5 - 126.9) | 145.9 (110.7 - 166.2) | 0.000* |
| HOMA-S | 97.5 (78.8 - 124.9) | 93.2 (73.9 - 115.8) | 83.8 (66.9 - 104.9) | 71.8 (57.5 - 87.3) | 45.3 (40.0 - 56.2) | 0.000* |

| | | | | | | |
|---|---|---|---|---|---|---|
| Compensatory $\beta$-cell response | 83.2 (70.6 - 103.6 ) | 83.9 (70.8-102.4) | 79.6 (67.9 - 95.4) | 71.8 (61.9 - 90.1) | 66.4 (53.2 - 78.1) | 0.000* |

**Legends for Supplementary Table** 2: Median ($25^{th}$-$75^{th}$ centile) or n (%); * statistically significant ($p<0.05$) by Kruskal-Wallis Test for continuous data and Chi square test for categorical data; Q1, Q2, Q3, Q4 are quartiles of waist circumference Q4 is further divided into 2 groups of (normal) and (centrally obese)

**Supplementary Figure 1:** Glycemic parameters of PD and NGT adolescent girls across fat% quartiles.

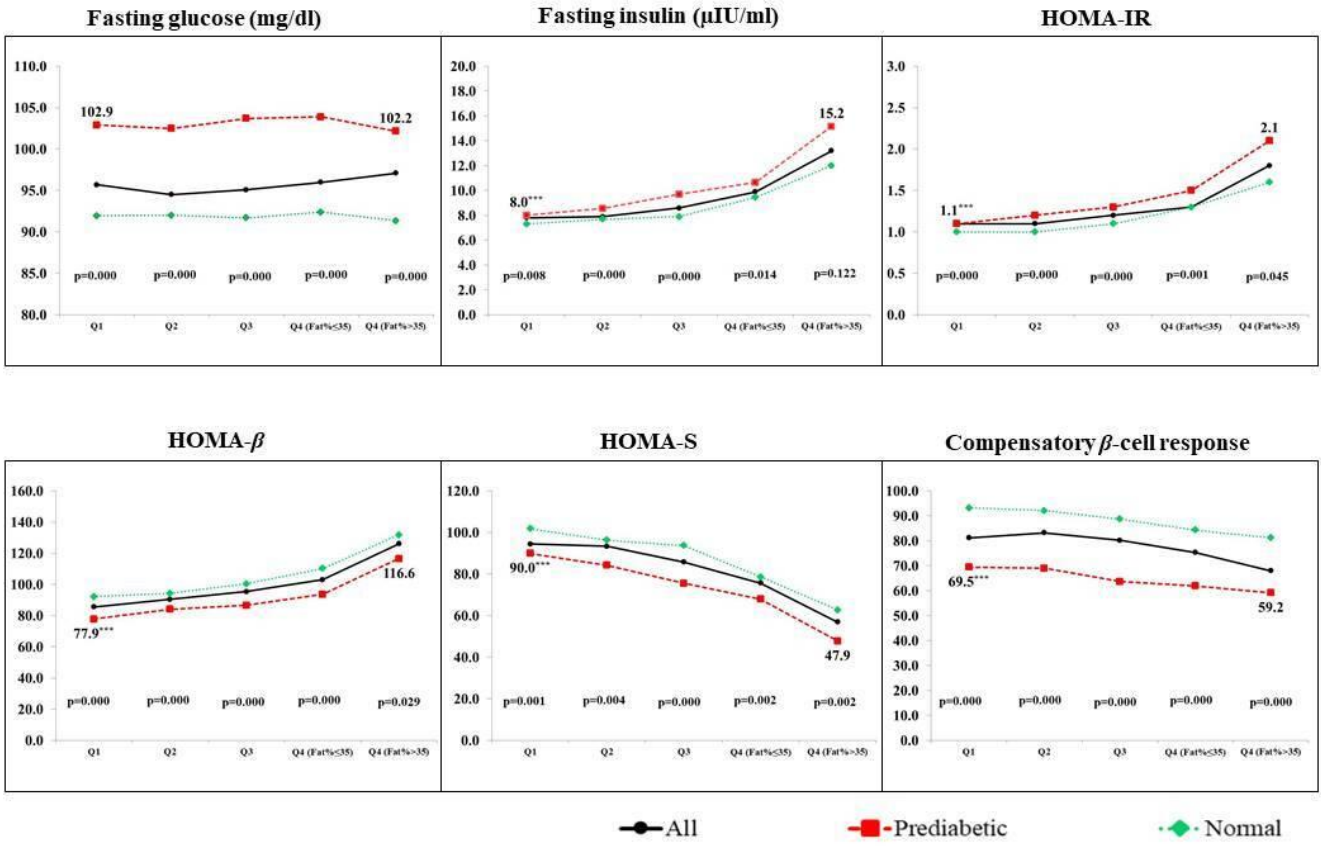


**Legends for Supplementary Figure 1:** Q1, Q2, Q3, Q4 are quartiles of body fat%. Q4 is further divided into 2 groups of (Fat% ≤ 35) and (Fat% >35); p- p value by Kruskal-Wallis Test

Note: Data represented by median values.

**Supplementary Figure 2:** Glycemic parameters of PD and NGT adolescent girls across waist circumference quartiles.

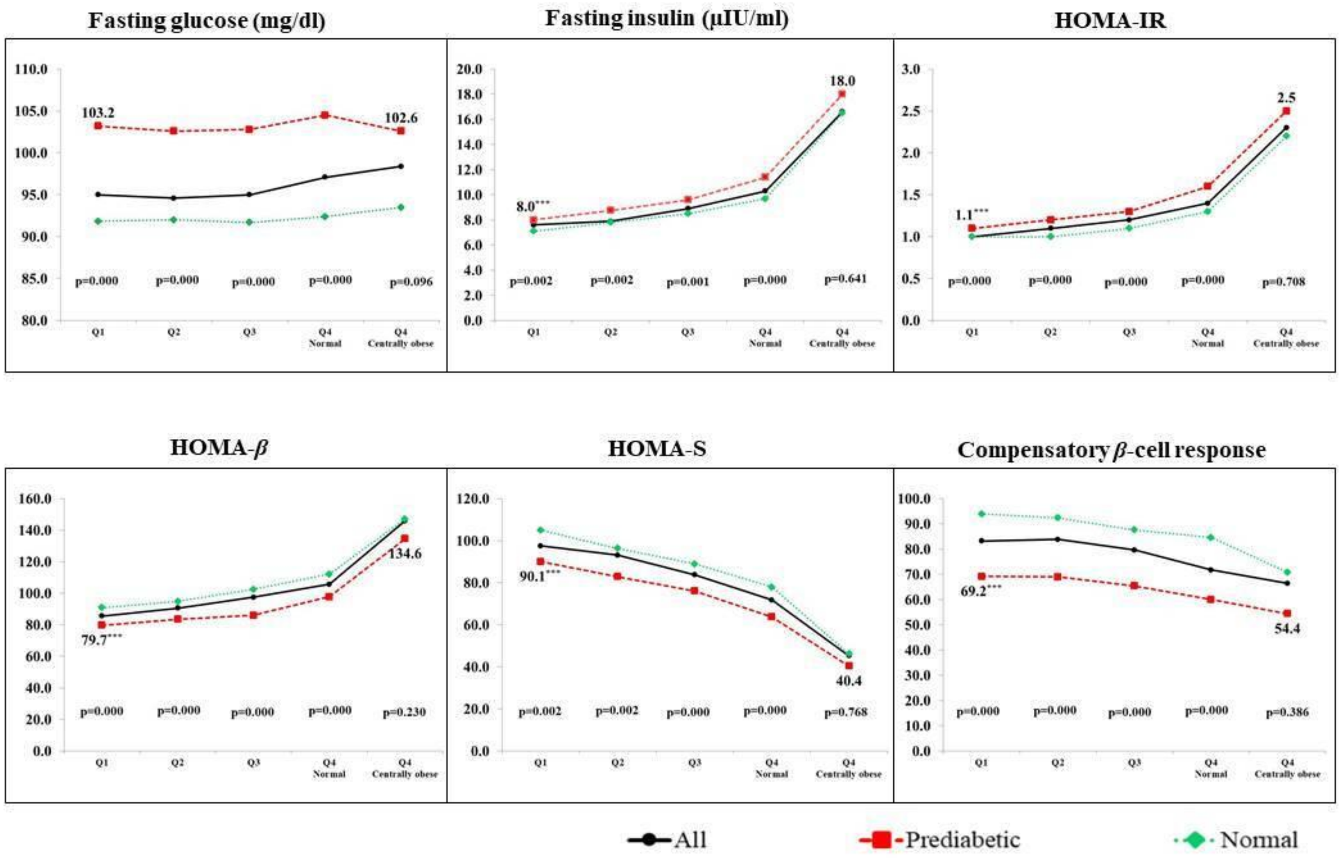


**Legends for Supplementary Figure 2:** Q1, Q2, Q3, Q4 are quartiles of waist circumference Q4 is further divided into 2 groups of (normal) and (centrally obese); p- p value by Kruskal-Wallis Test

Note: Data represented by median values.
